# Infants predict expressers’ cooperative behavior through facial expressions

**DOI:** 10.1371/journal.pone.0185840

**Published:** 2017-10-04

**Authors:** Toshinori Kaneshige, Etsuko Haryu

**Affiliations:** Graduate School of Education, University of Tokyo, Bunkyo-ku, Tokyo, Japan; Universita degli Studi di Udine, ITALY

## Abstract

This study investigated infants’ ability to use facial expressions to predict the expressers’ subsequent cooperative behavior. To explore this problem, Experiment 1 tested 10- and 14-month-olds (N = 16, respectively) by using a violation-of-expectation procedure. In the experiment, all infants were first familiarized with two models, one with a happy facial expression and the other with an angry expression. They were also familiarized with an event in which a duck puppet tried to open a box but failed. During the test phase, infants in the helping condition saw two test scenes; one in which the happy model helped the duck open the box, and the other in which the angry model helped the duck. Infants in the hindering condition saw a test scene in which the happy model hindered the duck and the other test scene in which the angry model hindered the duck. The results demonstrated that both 10- and 14-month-olds looked longer at the angry model than at the happy model in the helping condition, whereas they looked at the happy model as long as the angry model in the hindering condition. Experiment 2 tested 6-month-olds (N = 16) with a slightly modified procedure and found the same tendency as shown by 10- and 14-month-olds. These results suggest that infants as early as at 6 months do not predict that a person with an angry expression will help others. However at the same time, they do not clearly understand the incongruence between happy expressions and hindering behavior. The results were discussed by referring to a negativity bias and human environment in which infants grow up.

## Introduction

Facial expressions convey information about the expresser’s inner states and personality traits [1–3]. For example, if we see a person with a happy face, we think that s/he is ready to communicate with others, and we do not hesitate to talk to her/him. In contrast, if we see a person with an angry face, we think that s/he does not feel like interacting with others in a positive way, and we are unwilling to communicate with her/him.

Developmental studies have revealed that in their first year, infants showed ability to understand facial expressions. Neonates, not only full-term but also pre-term, discriminate facial expressions presented by a live actor [4,5]. Infants at 4 months categorize facial expressions. That is, they begin to identify sameness in facial expressions, for instance, happiness across different people [6–8]. By 6 months of age, infants begin to show rudimentary understanding of facial expressions’ emotional values: They show more positive responses to happy faces than to angry faces [6,8]. Seven-month-olds can match a facial expression to its corresponding vocal tone [9–12]. Infants at 12 months can use others’ emotional expressions to decide how they behave in an ambiguous situation (“social referencing,” [13]).

Moreover, there are a handful of studies investigating whether infants use others’ facial expression to predict the expresser’s behavior [14–17]. Barna and Legerstee [14] investigated this problem using a violation-of-expectation procedure comprising three phases. During the first phase, 9-month-olds saw two events alternately. In one event, model A looked at a ball with happy facial and vocal expressions; in the other event, model B looked at the ball with unhappy emotional expression. During the second phase, infants were habituated to a movie in which the ball was held by a person whose face was occluded by the curtain. In the test phase after the habituation, infants were shown two test scenes. In one test scene, the model A who had shown happy emotional expression toward the ball held the ball, and in the other test scene, the model B who had shown unhappy emotional expression toward the ball held the ball. The analyses revealed that the infants were more surprised when seeing the unhappy model holding the ball than seeing the happy model. The results suggest that 9-month-olds do not predict that a person should reach for the object toward which s/he showed unhappy emotional expression.

By measuring infants’ pupil dilation, another study has revealed that 10- and 14-month-olds use happy and angry facial expressions to predict expressers’ behaviors toward an object [15]. In this study, infants were shown two types of movies. In one type (congruent movies), a model with a happy emotional expression (face and voice) gently stroked a stuffed tiger, or a model with an angry emotional expression thumped the tiger with a closed fist. In the second type of movie (incongruent movies), a model with a happy expression thumped the tiger, or a model with an angry expression gently stroked the tiger. Results demonstrated that both 10- and 14-month-olds showed more pupil dilation when they saw the model with an angry expression gently stroking the tiger than when they saw the model with the same emotional expression thumping the tiger. Furthermore, 14-month-olds increased pupil dilation when they saw the happy model thumping the tiger compared to when they saw the same model gently stroking the tiger. Thus, this study indicated that infants at 10 months predicted the behavior of a person with angry emotional expression, and by 14 months, infants also predicted the behavior of a person with a happy emotional expression.

As seen so far, previous studies have revealed that before their first birthdays, infants begin to predict the expresser’s behavior toward an object through facial expressions. However, whether infants predict the expresser’s social behavior through facial expressions remains unknown. When we communicate with a person, information about how that person would behave toward others is very important. In particular, the ability to distinguish reliable, cooperative people from uncooperative people is essential for human life in which interaction with others is inevitable [18–22]. In fact, the literature has shown that adults use facial expressions to decide whether the expresser is reliable or not: Adults regard people with happy faces as reliable and friendly, and those with angry faces as hostile and unfriendly [23,24]. When do infants begin to use facial expressions to predict others’ cooperative behavior?

The present study investigated this problem by using a violation-of-expectation procedure and testing 10- and 14-month-olds, who have already been reported to understand the relationship between facial expressions and behavior toward an object. In the experiment, half the infants were assigned to the helping condition, and the other half to the hindering condition. More specifically, we first familiarized infants with two models, Model A with a happy facial expression and Model B with an angry facial expression (model-learning phase). After this familiarization, we also familiarized the infants with an event in which a duck (puppet) tried to open a transparent box to retrieve a toy, but finally failed (event-learning phase). During the test phase after these two familiarization phases, the infants were shown two test events, one consistent and the other inconsistent. In the helping condition, infants saw that Model A with a happy facial expression helped the duck open the box (consistent test event) in happy-model test trials and that the Model B with an angry facial expression helped the duck (inconsistent test event) in angry-model test trials. In the hindering condition, infants were shown movies in which Model B with an angry facial expression hindered the duck from opening the box (consistent test event) in angry-model test trials, and in which the happy model hindered the duck (inconsistent test event) in happy-model test trials. If infants were to predict models’ cooperative behavior using models’ facial expressions, they should look longer at the inconsistent test event than at the consistent test event.

## Experiment 1

### Methods

#### Participants

Sixteen healthy, full-term 10-month-olds (8 boys and 8 girls, *M* = 10 months 14 days, *SD* = 10 days) and 16 healthy, full-term 14-month-olds (8 boys and 8 girls, *M* = 14 months 15 days, *SD* = 6 days) participated in the experiment. An additional twenty-one 10-month-olds and fifteen 14-month-olds were excluded from analysis because of fussiness (16 infants), not looking at the critical scene during test trials (14 infants), experimenter error (2 infants), and equipment error (4 infants). Infants were recruited through public notices or Internet and were given a token for participation. All infants lived in the Greater Tokyo area. Half were assigned to the helping condition and half to the hindering condition. This experiment was approved by the ethical committee of the Graduate School of Education, University of Tokyo. All caregivers provided their written informed consent before the experiment. The experiment was conducted from October 1, 2013, to May 18, 2015.

#### Stimuli

**Fig 1:**
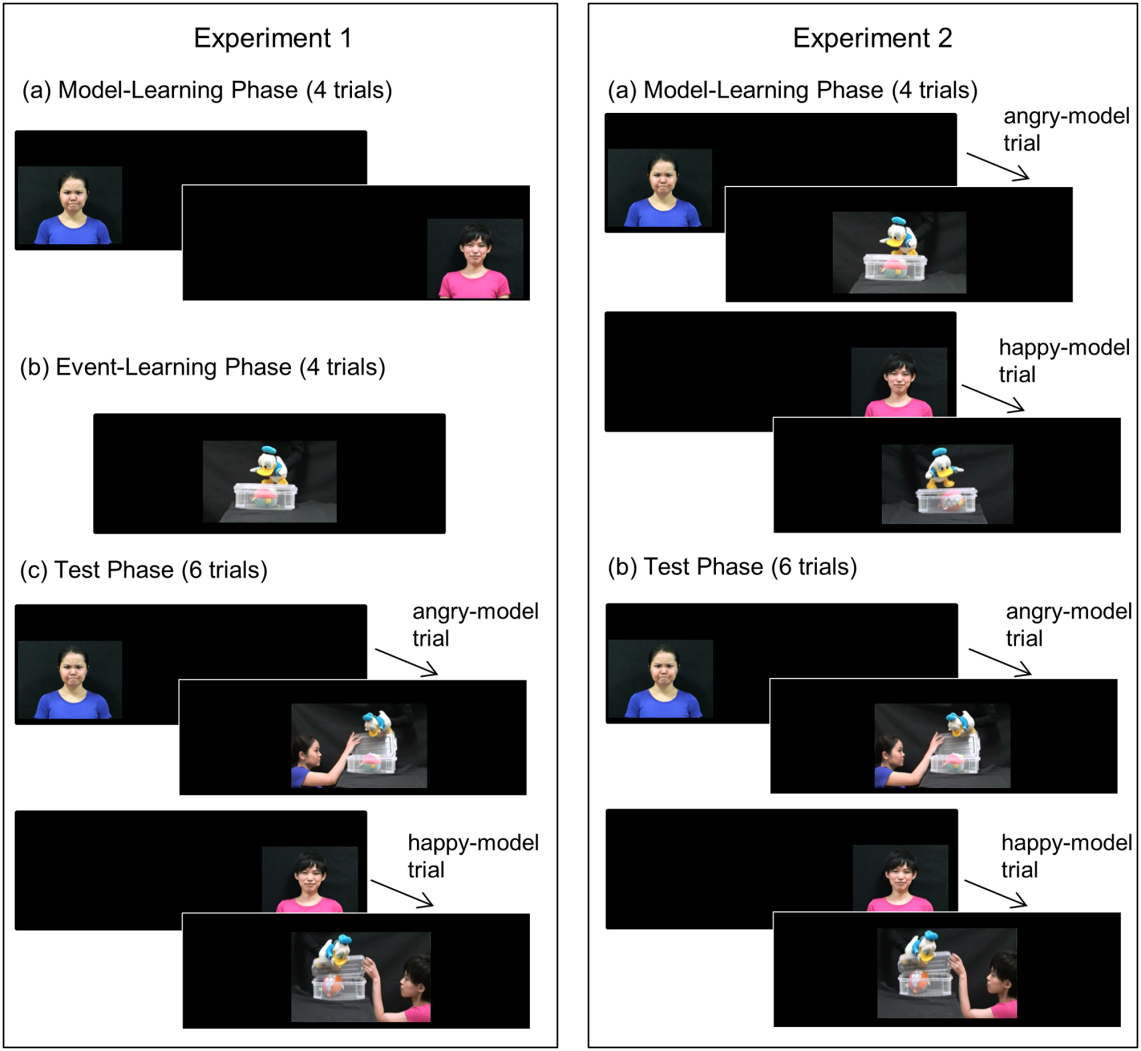
Illustration of the procedure in Experiments 1 and 2 (in the helping condition). Experiment 1: (a) During the model-learning phase, infants saw two models, each with a predetermined facial expression. (b) During the event-learning phase, infants were presented with a movie in which a duck puppet twice tried to open a box but failed. (c) During the test phase, after infants saw a model with the predetermined facial expression on the side monitor, they were presented with a test-event movie on the central monitor. Experiment 2: (a) During the learning phase trials, infants successively saw a model movie and an event movie in a trial. (b) During the test phase, immediately after infants saw the model present a facial expression on the predetermined side monitor, they saw the test event movie on the central monitor.

The experiment consisted of three phases, model-learning, event-learning, and testing (Fig 1, left column). We developed movie stimuli for each phase’s presentation (S1, S2 and S3 Movies). More specifically, in the model-learning phase, infants were familiarized with two models, one with a happy smiling face and the other with an angry face. To develop these movies (model movies), we asked two Japanese females (24 and 28 years old) to present a happy face and an angry face. The models were instructed to remove their earrings and glasses and to practice happy and angry expressions in a mirror, imitating samples of these expressions from Ekman and Friesen’s study [25]. We video-recorded models shifting their facial expressions from a neutral to a happy or an angry expression, and created 10-second-long movie which showed a model shifting her facial expression for the first second and kept showing the resultant facial expression for the following 9 seconds.

For the event-learning phase, we filmed a scene in which a duck (a hand puppet) twice tried to open a transparent box to retrieve a colorful ball but finally failed. A black curtain covered the stage and background. This event movie was constructed in two versions: In one version, the duck appeared on the box’s left side; in the other, it appeared on the box’s right side. We constructed these two movies to let the infants know that the puppet would appear on both sides of the box during the test phase. Each movie was 10 seconds long.

For the test phase, two types of test-event movies were constructed: One showed a model helping the duck open the box, and the other, a model hindering the duck. In the stimulus movie of helping behavior, a model first entered the scene from one side of the stage, and saw the duck twice try to open the box and fail. When a model entered from the left, the duck first appeared on the box’s right side on the central monitor. When a model entered from the right, the duck appeared on the box’s left side. (As described above, the infants had already been presented the two scenarios in which the duck appeared on the box’s left and right sides.) Then, on the duck’s third attempt, the model helped the duck open the box by lifting the box’s lid with the duck; as a result, the duck retrieved the ball. In the stimulus movie of hindering behavior, a model first entered the scene from one side of the stage. After observing the duck twice try and fail to open the box, the model swung her hand down on and pressed the box’s lid so that it would not open.

In the test-event movies, the model kept showing a neutral face, because we would like to examine not whether infants could *match* facial expression to likely behavior, but rather whether they could use facial expressions to *predict* the expresser’s subsequent behavior. In fact, it was difficult even for adults to tell what facial expression models showed in the test-event movies, because they kept showing their face in profile (see S2 Dataset). For the model to finish helping or hindering the duck took 6 seconds, and the movie showing the resultant scene lasted for a further 30 seconds. The models in this manuscript has given written informed consent (as outlined in PLOS consent form) to publish these case details (in Fig 1 and Supporting Information).

#### Apparatus

**Fig 2:**
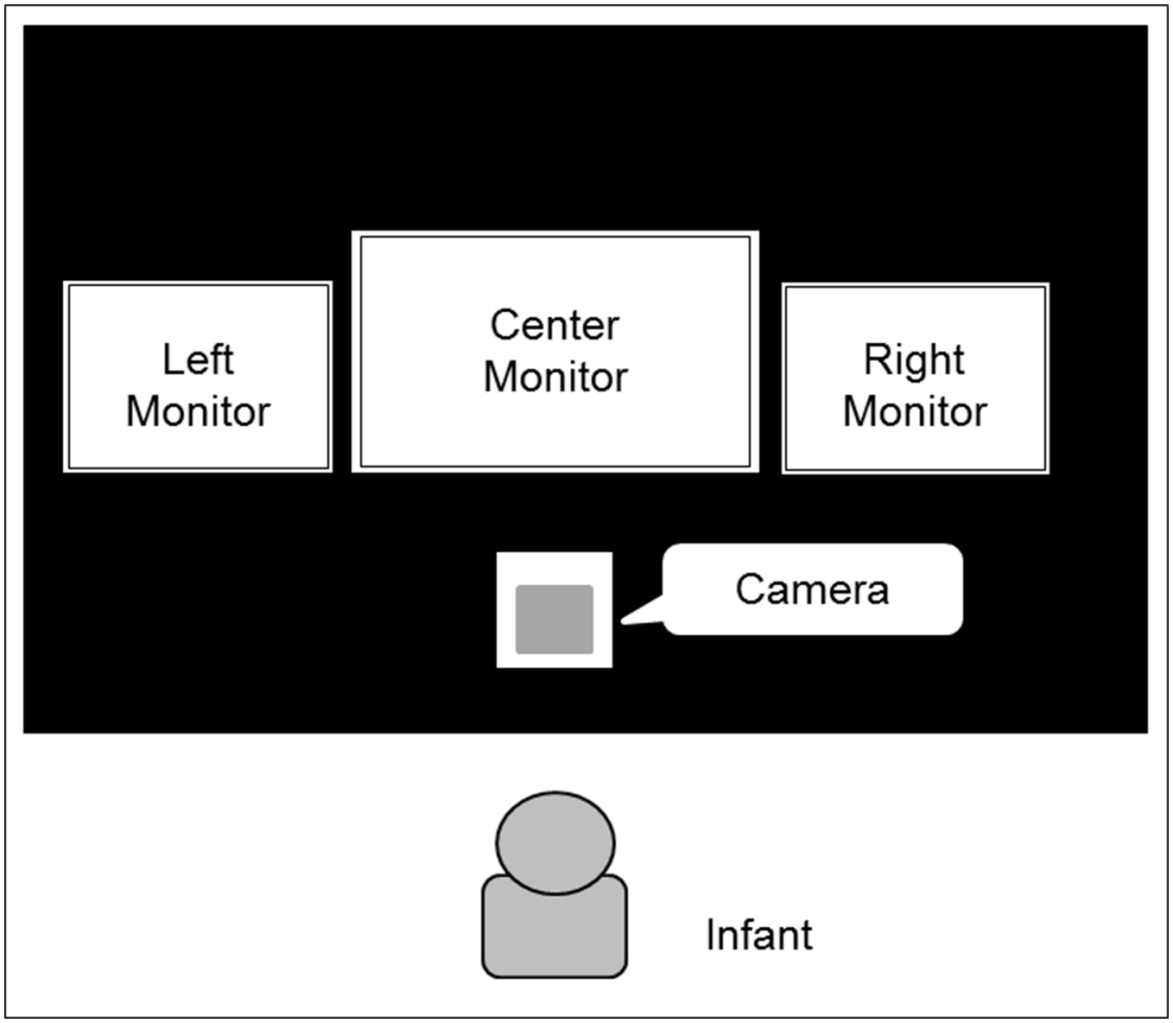
Apparatus of experiments.

Fig 2 depicts the apparatus. There were three monitors, one 17-inch monitor in the center and two 15-inch monitors on the left and right sides. These three monitors were aligned horizontally without gap from each other. An infant was seated on a parent’s lap 60 cm back from the central monitor. Below the central monitor, a digital video camera was concealed and recorded infant’s looking behavior during stimulus presentation at 30 frames per second. The experimenter could observe the infants’ looking behavior from an adjacent room.

#### Procedure

Before starting the experiment, caregivers were instructed not to look at the monitors and not to interact with the infant during the experiment. We used a violation-of-expectation paradigm and the presentation of stimuli was controlled by Habit X 1.0 [26]. A movie of a revolving plate was shown on the central monitor in the inter-trial interval. Once the infant looked at the central monitor, the experimenter pressed a computer key to turn off the revolving plate and start a trial. As shown in Fig 1 (left column), the experiment consisted of three phases: (a) the model-learning phase, (b) the event-learning phase, and (c) the test phase. The number of trials during each phase was predetermined.

(a) Model-Learning Phase: Infants were presented with two models, each presenting different facial expressions. The number of trials in this phase was fixed to four. In each trial, infants saw one model presenting a particular facial expression on either the left or the right monitor. Which model posed a happy expression or an angry expression was fixed for each infant. That is, half of infants saw Model A with a happy expression and Model B with an angry expression. The remaining half saw Model A with an angry expression and Model B with a happy expression. The monitors’ left-right position in which each model appeared was fixed for all infants: Model A always appeared on the left monitor, and Model B always on the right. Infants were presented with the stimulus movie twice alternatively for each model. Each model’s presentation order was counterbalanced across infants. The length of one trial was fixed at 10 seconds. During the 10-second movie, the model took 1 second to shift her face to a predetermined expression and then maintained the facial expression during the remaining 9 seconds.

(b) Event-learning Phase: Immediately after the model-learning phase, the event-learning phase began. The stimulus movies in which a duck (puppet) twice tried and failed to open a box was presented on the central monitor. The event-learning phase had 4 trials, and infants saw two versions of the stimulus movie twice alternatively (first, the duck appearing on the box’s left side, and second, the duck appearing on the right side). Each trial ended when 10 seconds had elapsed. The stimulus movies’ presentation order depended on the models’ presentation order during the model-learning phase. That is, infants who had seen the left monitor during the model-learning phase’s first trial saw the movie in which the duck appeared on the box’s right side during the event-learning phase’s first trial and vice versa. The combination of a model on the left monitor and an event movie in which the duck appeared on the box’s right side was the sequence with which the infants were shown those movies during the test phase.

(c) Test Phase: In each test-phase trial, infants successively saw the model and the event-test movie. More specifically, infants first saw the same model with the same facial expression on the same (left or right) monitor as in the model-learning phase for 3 seconds. Immediately after the monitor in which the model appeared was turned off, the test-event movie was presented on the central monitor. Infants in the helping condition saw the test-event movie of helping behavior, and infants in the hindering condition saw the test-event movie of hindering behavior. Therefore, an infant in the helping condition, for example, first saw Model A with an angry face on the left monitor for 3 seconds. Immediately after the left monitor was turned off, the test event movie in which Model A entered the scene from the left side, observed the duck twice fail to open the box and then helped the duck’s third try be successful. The model and duck stopped moving and remained still when the duck retrieved the goal toy after they succeeded in opening the box. A trial ended when 39 seconds had elapsed (3 seconds for stimuli of facial expressions and 36 seconds for test stimuli).

The test phase had 6 trials, and thus infants underwent three Model-A and three Model-B trials alternatively. The models’ presentation order during the test phase was the same as during the model-learning phase. That is, infants who saw Model A on the left monitor during the model-learning phase’s first trial, and who had seen the movie where the duck appeared on the box’s right side during the event-learning phase’s first trial also saw Model A posing with a facial expression on the left monitor during the test phase’s first trial. After that, s/he saw that the puppet appeared on the right side of the box and Model A entered the scene from the left side. Infants who looked away before the test-event movie’s critical scene (from the frame in which models began to move their hands, to the frame in which models’ hands left the box’s lid) were excluded from the final sample.

#### Coding

Infants’ looking times during the test phase were measured off-line by an observer blind to stimuli presented to infants. More specifically, infants’ looking was timed starting when the duck’s trial ended in success or failure until the stimulus presentation ended after a 30-second long still scene. The number of frames during the last 30 seconds of the stimuli presentation was counted and the looking times were calculated. The six test trials consisted of three happy-model trials in which the happy model helped or hindered the duck and three angry-model trials in which the angry model helped or hindered the duck. Therefore, average looking times across three trials were calculated for happy-model and angry-model test trials, respectively. To establish inter-observer reliability, a second coder also judged infants’ looking off-line for approximately 30% of participants. The correlation coefficient was r = .98.

### Results and discussion

**Fig 3:**
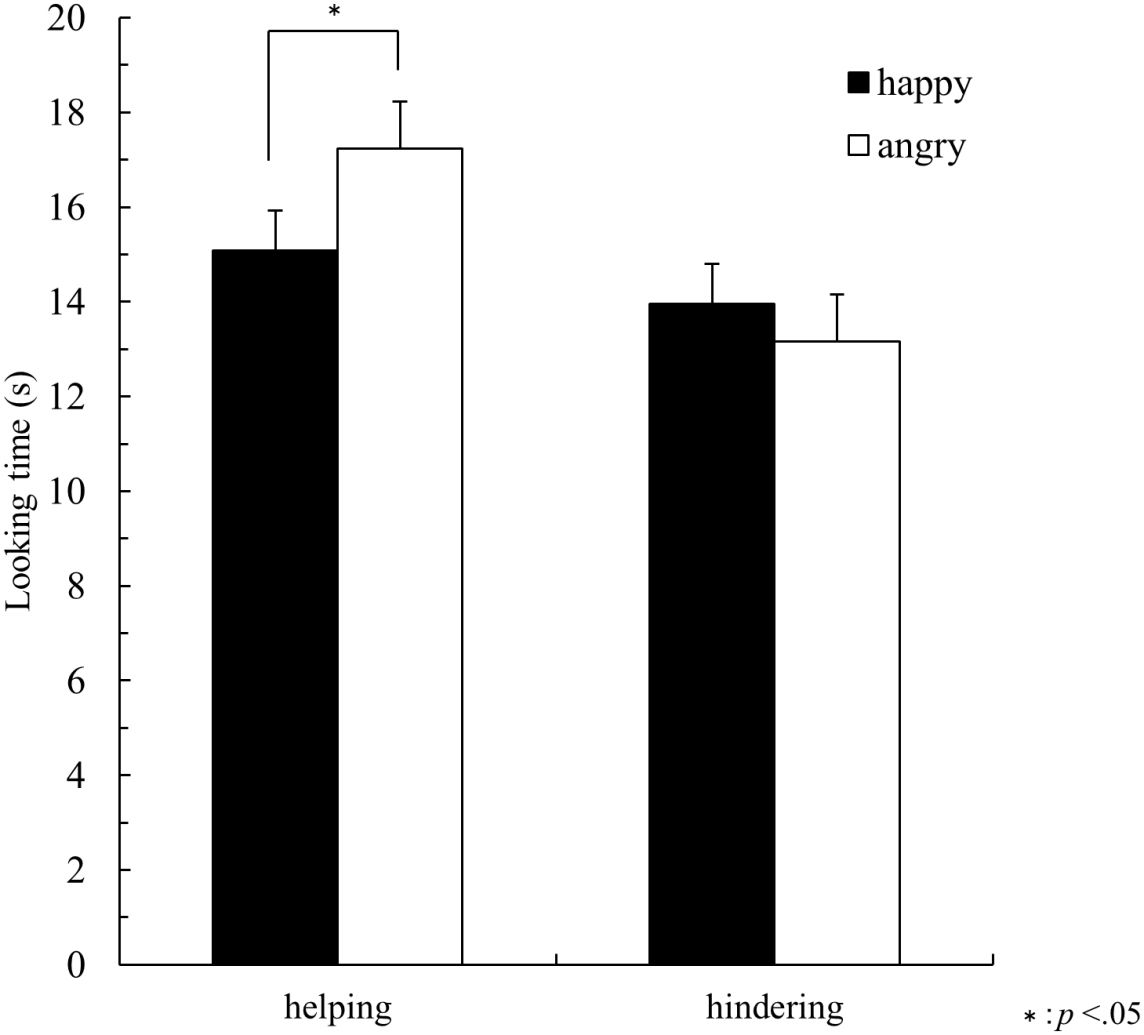
Mean looking times shown by 10- and 14- month-olds (collapsed across age) on happy-model and angry-model test trials in Experiment 1 (Mean + SE).

Fig 3 shows looking times averaged across three happy-model and three angry-model test trials. For 10-month-olds in the helping condition, mean looking times were 14.76 seconds (*SD* = 2.32) for happy-model test trials and 16.22 seconds (*SD* = 2.95) for angry-model test trials; in the hindering condition, mean looking times were 15.04 seconds (*SD* = 2.88) for happy-model test trials and 14.34 seconds (*SD* = 3.83) for angry-model test trials. For 14-month-olds in the helping condition, mean looking times were 15.40 seconds (*SD* = 5.24) for happy-model test trials and 18.25 seconds (*SD* = 3.69) for angry-model test trials; in the hindering condition, mean looking times were 12.85 seconds (*SD* = 2.04) for happy-model test trials and 11.97 seconds (*SD* = 5.12) for angry-model test trials. Looking times were submitted to a 2×2×2 analysis of variance (ANOVA) with age group (10 m, 14 m) and condition (helping or hindering) as a between-participants factor and trial (happy-model or angry-model test trials) as a within-participants factor.

ANOVA revealed significant interaction between condition and trial, *F* (1, 28) = 5.36, *p* = .03, *η*_*p*_^*2*^ = .16. Post hoc pairwise comparisons indicated that infants in the helping condition showed significantly longer looking times at the angry model (*M* = 17.23, *SD* = 3.28) than at the happy-model (*M* = 15.08, *SD* = 3.80). In the hindering condition, infants looked at the happy-model (*M* = 13.95, *SD* = 2.58) as long as at the angry model (*M* = 13.16, *SD* = 4.39). Furthermore, infants in the helping condition looked at the angry model longer than infants in the hindering condition, whereas infants in the helping condition looked at the happy model as long as infants in the hindering condition.

Results indicate that 10- and 14-month-olds regard the combination of an angry face and helping behavior as inconsistent and unexpected compared to the combination of a happy person and helping behavior. On the other hand, they did not react differently when they observed a person with a happy face hinder the duck than when they observed an angry person hinder the duck. The present study thus found that 10-month-old infants, as well as 14-month-old infants, did not predict that a person with an angry face would help others. This is the age at which previous studies have reported that infants use facial expressions to predict the expresser’s behavior toward an object [14,15]. At the same time, results suggested neither 10- nor 14-month-olds understand the incongruence between happy facial expressions and hindering behavior. This tendency, in which infants are more sensitive to signs that might indicate a negative consequence than to those that might indicate a positive consequence is also consistent with findings in previous studies that suggested infants’ negativity bias [27]. The negativity bias is the tendency that infants possess to use negative information earlier than positive information. For example, infants at 3 months of age avoid harmful agents compared with neutral agents, whereas they do not prefer helpful agents to neutral agents [21]. Studies testing infants’ ability to understand facial expressions also have revealed that 10-month-olds were surprised at the angry-faced model gently stroking a doll, whereas they were not surprised at the happy-faced model thumping the doll [15]. This bias that infants understand negative information earlier than positive information was again found in the present study by testing infants’ ability to understand the relationship between others’ facial expressions and their cooperative behavior. We will return to this problem in the General Discussion.

Experiment 1 demonstrated that 10- and 14-month-old infants are already able to use facial expressions to predict the expressers’ cooperative behavior. However, Hamlin and colleagues [21], testing infants’ understanding of cooperative behavior, showed that even 3-month-olds responded differently to a helpful agent than to an uncooperative agent after observing agents’ helping or hindering behavior. Furthermore, previous studies have found that infants begin to react appropriately to the emotional valence of facial expressions from at least 6 months onward [6,8]. Considering these findings, we might predict that infants as young as 6 months may also understand the relationship between facial expressions and the expresser’s cooperative behavior.

To investigate 6-month-olds’ ability, we used the same stimuli and procedure as in Experiment 1. However, the infants became bored easily and looked away before the movie showed the critical scene, especially during the test phase. Therefore, we modified the procedure of Experiment 1, and conducted Experiment 2 for 6-month-olds.

## Experiment 2

The aim of Experiment 2 is to investigate whether 6-month-olds, who have just begun to understand the affective meanings of facial expressions, also use facial expressions to predict the expresser’s cooperative behavior. In Experiment 2, we modified the procedure of Experiment 1 to test 6-month-olds. That is, we reduced the number of trials, shortened the trial length, and modified the experimental structure from three phases to two: a learning phase and a test phase (see Fig 1, right column).

### Method

#### Participants

Sixteen healthy, full-term 6-month-olds (9 boys and 7 girls, *M* = 6 months 12 days, *SD* = 8 days) participated in the experiment. An additional eleven 6-month-olds were excluded from analysis because of fussiness (1 infant), not looking at the test phase’s critical scene (8 infants), and experimenter error (2 infants). Infants were recruited through public notices or Internet and were given a token for participation. All infants lived in the Greater Tokyo area. Half the infants were assigned to the helping condition, and the other half to the hindering condition. All caregivers provided their written informed consent before the experiment. The experiments were conducted from October 2, 2014, to September 28, 2015.

#### Stimuli

The experiment consisted of two phases; the learning phase and the test phase. In one trial during the learning phase, infants first saw a model present a predetermined facial expression and move toward the central monitor (model movie). Then the central monitor was turned on, and showed the duck try to open the box, but fail twice (event movie). For event movies, we used the same stimuli as in Experiment 1. Model movies were newly constructed so that they would more likely attract infants’ attention. That is, in the model movie, over the first second, the model shifted her facial expression from neutral to a predetermined (happy or angry) expression, held the facial expression for one second, took another second to return her facial expression to neutral, and then moved toward the central monitor. Thus, the model movie lasted 4 seconds. Two versions of the model movie (with happy or angry facial expressions) were filmed for each of the same two models as in Experiment 1.

For the test phase, the test-event movies used in Experiment 1 were shortened. The scene in which the duck twice tried to open the box before a model helped or hindered him was removed. As a result, in the first 3 seconds of the test-event movie, the model helped or hindered the duck’s first attempt, and the movie lasted for a further 30 seconds.

#### Apparatus

The apparatus for Experiment 2 was identical to that of Experiment 1.

#### Procedure

In Experiment 2, the model-learning phase and the event-learning phase from Experiment 1 were combined into the learning-phase. Thus, Experiment 2 had two phases: (a) the learning phase and (b) the test phase. Although trial length was fixed during the learning phase, stimuli presentation was infant-controlled during the test phase. As in Experiment 1, the left–right position in which each model was presented was fixed throughout the experiment.

(a) Learning phase: In each trial during this phase, infants were successively presented with a model movie and an event movie. More specifically, infants were first presented with a model movie on either the left- or right-side monitor, which showed a female model presenting a predetermined facial expression, returning her face to neutral, then moving toward the central monitor, and disappearing from the side monitor. Immediately after the side monitor was turned off, the event movie began on the central monitor. In the event movie, the duck appeared on the box’s opposite side from the monitor in which the model had appeared and twice tried and failed to open the box. Trial length was fixed to 14 seconds (4 seconds for a model movie, plus 10 seconds for the event movie). The relation between a model and a facial expression (happy or angry) was fixed by infant. This phase had 4 trials, and infants saw each model twice alternately. Each model’s presentation order was counterbalanced across infants.

(b) Test phase: In each test-phase trial, infants successively saw the model movie and the test-event movie. More specifically, infants first saw a model with the same facial expression on the same (left or right) monitor for 4 seconds, as in the learning phase. As soon as the model disappeared from the side monitor, the test-event movie was presented on the central monitor. Infants in the helping condition saw the test-event movie of helping behavior, and infants in the hindering condition saw the test-event movie of hindering behavior. Therefore, an infant in the helping condition, for example, first saw Model A with an angry face on the left monitor for 4 seconds. Immediately after the left monitor was turned off, the test-event movie in which Model A helped duck’s try to be successful was presented. When the duck successfully retrieved the goal toy, the movie went still. A test-phase trial ended when the infant looked away from the monitor for 1.5 seconds or 37 seconds had elapsed (4 seconds for facial-expression stimuli, plus 33 seconds for the test stimuli).

The test phase had 6 trials, and thus infants underwent three Model-A trials and three Model-B trials alternatively. The models’ presentation order depended on the models’ presentation order during the learning phase: An infant who was presented with Model A first during the learning phase was also presented with Model A first during the test phase. Infants who looked away before the test-event movie’s critical scene (from the frame in which models began to move their hands, to the frame in which models’ hands left the box’s lid) were excluded from the final sample.

#### Coding

Infants’ looking times during the test phase were measured off-line by an observer in the same manner as in Experiment 1. The second coder also judged infants’ looking off-line for approximately 30% of participants, and the correlation coefficient was *r* = .97.

### Results and discussion

**Fig 4:**
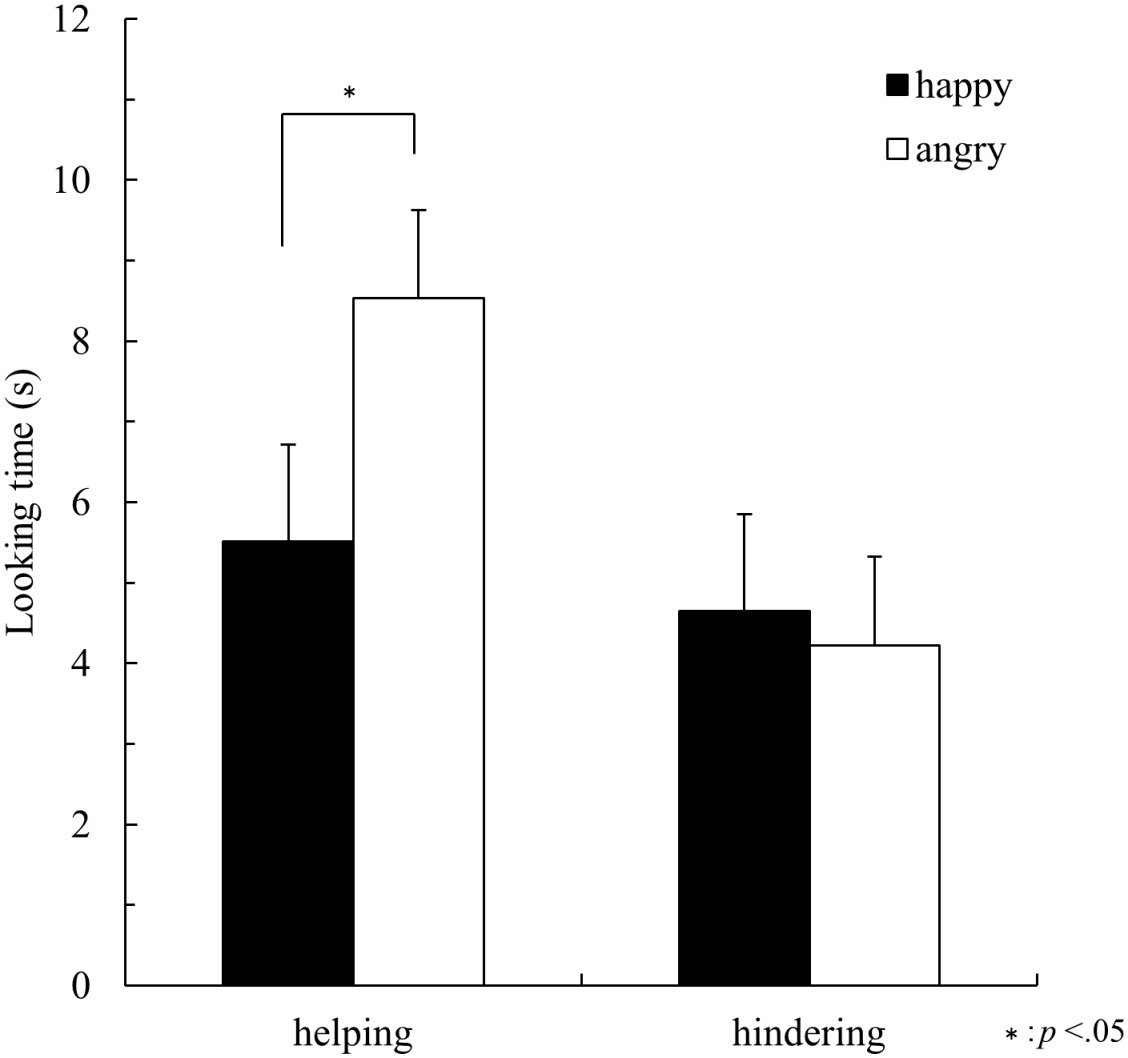
Mean looking times shown by 6-month-olds on happy-model and angry-model test trials in Experiment 2 (Mean + SE).

Fig 4 shows mean looking times on happy-model and angry-model test trials. In the helping condition, mean looking times were 5.51 seconds (*SD* = 3.87) for happy-model test trials and 8.53 seconds (*SD* = 4.01) for angry-model test trials. In the hindering condition, mean looking times were 4.65 seconds (*SD* = 2.77) for happy-model test trials and 4.22 seconds (*SD* = 2.46) for angry-model test trials. A 2×2 analysis of variance (ANOVA) with condition (helping, hindering) as a between-participants factor and trial (happy-model, angry-model test trials) as a within-participants factor was conducted.

ANOVA revealed significant interaction between condition and trial (*F* (1, 14) = 5.80, *p* = .03, *η*_*p*_^*2*^ = .29). Post hoc pairwise comparisons indicated that infants in the helping condition looked longer at the angry model than at the happy model. In the hindering condition, infants looked at the happy model as long as at the angry model. Furthermore, infants in the helping condition looked at the angry model longer than infants in the hindering condition, whereas infants in the helping condition looked at the happy model as long as infants in the hindering condition.

Thus, 6-month-olds in Experiment 2 showed the same tendency as 10- and 14-month-olds in Experiment 1. That is, 6-month-olds regarded the combination of an angry person and the helping behavior as inconsistent and unexpected, compared to the combination of a happy person and helping behavior. On the other hand, they looked at the scene in which a person with a happy face hindered the duck as long as at the scene in which an angry person hindered the duck. Compared to previous studies that found 10-month-olds’ ability to use facial expressions [14,15], 6 months was earlier. Thus, the present study for the first time found that infants as young as 6 months were able to use facial expressions to predict the expresser’s subsequent cooperative behavior.

## General discussion

The present study investigated whether infants at 6, 10, and 14 months use facial expression to predict the expresser’s subsequent cooperative behavior. Results showed that infants in all age groups looked significantly longer at helping behavior by a person with an angry face than at helping behavior by a person with a happy face, suggesting that even at 6 months, infants do not predict that a person with an angry face will help others. On the other hand, infants in all age groups looked at hindering behavior by the happy-faced model as long as at hindering behavior by the angry-faced model. This indicates that infants aged 14 months have not yet learned whether a person with an angry face or one with a happy face is more likely to hinder others.

However, there are two other possible explanations for the results. First, infants might have reacted to the novelty of the test event. In the present study, infants saw that the puppet always failed to open the box during the learning phase. Infants in the hindering condition saw again the duck fail to open the box during the test phase, whereas infants in the helping condition saw a novel event in which the box was successfully opened. The novelty of the outcome shown in the helping condition might have influenced infants’ looking behavior. If infants were surprised at the novelty of the outcome, the looking times of infants in the helping condition would be longer in both happy- and angry-model trials than those of infants in the hindering condition. However, the analyses revealed that infants in the helping condition looked at the happy model as long as infants in the hindering condition. The pattern of infants’ looking times indicated that infants reacted to the incongruence between the models’ anger expressions and helping behavior, not to the novelty of the outcome with the opened box.

The second possibility that should be considered is that infants perceived the models’ action as object-oriented behavior, not as cooperative behavior. That is, infants did not see the box-opening event as a social event but as an event in which the model just opened the box. But previous studies that examined infants’ social evaluation using the box-opening event suggested that even 5-month-olds regarded the event as social and person-oriented if and only if the agent helped or hindered an animate participant who had been trying to open the box. More specifically, 5- and 9-month-olds preferred a helper who helped an animate participant to a hinderer who hindered the animate participant, whereas they did *not* prefer a helper who “helped” an *inanimate object* (a pincer) by opening a box to a hinderer who “hindered” an *inanimate object* [19]. Although we did not examine whether infants’ looking was affected by the animate/inanimate status of the helped/hindered participant in the present experiments, the results from Hamlin and colleagues suggested that infants at 5 months of age saw opening the box together with an animate participant who has been trying to open it as a social event.

The results in the present study suggest that infants prepare for bad rather than for good things. On one hand, they think that either a happy-faced or an angry-faced person could behave uncooperatively. On the other hand, they specifically did not predict that a person with an angry face will help others. Such earlier understanding of angry faces than of happy faces has been found for 10-month-olds in a previous study [15]. In that study, 10-month-olds were surprised to see a person with an angry emotional expression gently stroking a stuffed tiger, but they were not surprised to see a person with a happy emotional expression thumping the tiger. This early sensitivity to angry faces can be regarded as a manifestation of negativity bias to social-emotional information [27]. The bias also appears in infants’ social evaluation. According to Hamlin et al. [21], 3-month-olds were attracted to prosocial agents rather than to anti-social agents, and they preferred neutral agents to antisocial agents; however, they did not prefer prosocial agents to neutral agents. That is, infants just avoid anti-social agents, rather than being attracted to prosocial agents. This tendency that infants possess to understand negative information earlier than positive information is the so-called negativity bias [27]. Given negativity bias, infants who already know facial expressions’ negative and positive values at 6 months [6,8] may more easily learn how a person with an angry rather than a happy expression is likely to behave.

In addition, infants’ daily interaction with their caregivers may also explain why infants’ understanding of an angry-faced person precedes that of a happy-faced person. In daily life, a caregiver with a happy face sometimes hinders an infant’s goal. For example, a caregiver may take away an object for which the infant is about to reach, because s/he is teasing the infant, wants to tell the infant that playtime is over, or thinks that the object is dangerous. On the other hand, when infants observe a person with an angry face, that person does not behave in a helpful way. Through such daily interaction, infants might more easily learn that a person with an angry face will not help others, but a person with a happy face is sometimes friendly and other times unfriendly.

There are at least four differences to be noted between the present research and previous studies that examined infants’ use of facial expressions to predict expressers’ behaviors. First, the present study examined infants’ understanding of the relationship between facial expressions and cooperative behavior, whereas Hepach and Westermann [15] focused on the relationship between facial expressions and behavior toward an object. Second, the present study found that even 6-month-olds can use an angry facial expression to predict the expresser’s uncooperative behavior; this is younger than in previous studies examining infants’ ability to predict the expresser’s behavior toward an object——most likely because previous studies tested only infants 9 months or older [13,14]. Therefore, if we tested whether 6-month-old infants would use facial expression to predict the expresser’s behavior toward an object, we might have found that they in fact had the ability. However, another possibility is that infants use facial expression to predict the expresser’s cooperative behavior earlier than to predict the expresser’s behavior toward an object. We cannot ignore this latter possibility since the ability to distinguish friends from enemies is essential for human life [18–22]. This problem should be investigated in future research.

Third, in the present research, infants were not surprised to see hindering behavior by a happy-faced person, even at 14 months. This may appear to contradict Hepach and Westermann’s [15] finding that 14-month-olds reacted more strongly when they saw a happy-faced person thumping a stuffed tiger, compared to when they saw the same happy-faced person gently stroking the tiger. This discrepancy might be explained by the nature of behavior examined. The present study focused on behavior toward a person, whereas Hepach and Westermann [15] treated behavior toward an object. The relationship between a happy facial expression and cooperative helping behavior might be more difficult to learn, compared to the relationship between a happy face and a gentle action toward an object for the following reason. On one hand, infants may sometimes observe a caregiver with a happy, smiling face hinder their intention, while at other times, they observe a happy-faced caregiver nursing, feeding, and calming them. That is, in interaction with infants, caregivers wear a happy face when helping infants as well as when they hinder infants’ intention. On the other hand, people may be likely to show a happy expression when they handle their favorite object, however they have no reason to smile at an object when they do not like it. Thus, given this distributional difference between a happy face that infants observe in association with cooperative behavior and one in association with an object-oriented behavior, the former relationship should be more difficult to learn than the latter.

Fourth, the present research showed infants soundless movies, which allowed us to examine their ability to use facial expressions per se. However, in natural settings, others’ emotional expressions are likely to be experienced not only with their faces but also with their voices. In fact, multimodal presentation of emotional expressions facilitates infants’ early recognition of emotions [28–30], and previous studies that investigated infants’ understanding of the relationship between facial expressions and object-oriented behavior presented infants with visual and aural emotional expressions [14–17]. It would be interesting to see whether multimodal presentation of emotional expressions would help infants to more successfully use the information, especially for happy faces, to predict other’s cooperative behavior.

To sum up, the present research has found 6-month-olds’ ability to predict others’ cooperative behavior based on prior facial expressions. Strictly speaking, whether infants infer the expresser’s current emotional states (i.e., the expresser is angry, so will not help others) or personality traits (i.e., the expresser is kind and friendly, so always helps others) based on facial expression remains unclear. However, the present research suggests that infants of at least 6 months of age use others’ facial expressions, especially angry faces, to prepare for the expresser’s (un)cooperative behavior.

## Supporting information

S1 Movie Model movies (four types of movie: happy and angry facial expression movies for each model). (MP4)

S2 Movie Movies that the puppet duck fails to open the box (two movies in which the duck appeared on the box’s left and right sides). (MP4)

S3 Movie Test-event movies (four types of movie: helping and hindering movies for each model). (MP4)

S1 Dataset Infants’ looking times in the Experiment 1 and Experiment 2. (XLSX)

S2 Dataset Adults’ responses in the additional research. To examine whether adults identify any emotion by looking at the models in profile in the test-event movie, we conducted additional research. Thirty-eight undergraduate students (mean age = 19.3, range = 19–20, 30 male and 8 female) participated in this research. All participants provided their informed consent before the research began and participated in a group. Participants were asked to label the models’ facial expressions freely using emotional words. Four types of test-event movies (2 models × 2 behaviors) were presented at the predetermined order. The presentation order of stimuli was decided by using randomly generated numbers. All test event movies were labelled as “no emotion” or “neutral” over 65.7% of the time (mean = 79.6, range = 65.7%–89.4%).
(XLSX)
